# Hyperbaric oxygen rapidly improves tissue-specific insulin sensitivity and mitochondrial capacity in humans with type 2 diabetes: a randomised placebo-controlled crossover trial

**DOI:** 10.1007/s00125-022-05797-0

**Published:** 2022-09-30

**Authors:** Theresia Sarabhai, Lucia Mastrototaro, Sabine Kahl, Gidon J. Bönhof, Marc Jonuscheit, Pavel Bobrov, Hisayuki Katsuyama, Rainer Guthoff, Martin Wolkersdorfer, Christian Herder, Sven G. Meuth, Sven Dreyer, Michael Roden

**Affiliations:** 1 Department of Endocrinology and Diabetology, Medical Faculty and University Hospital Düsseldorf, Heinrich-Heine-University, Düsseldorf, Germany; 2 Institute for Clinical Diabetology, German Diabetes Center (DDZ), Leibniz Institute for Diabetes Research at Heinrich-Heine-University, Düsseldorf, Germany; 3 German Center for Diabetes Research (DZD), Partner Düsseldorf, München-Neuherberg, Germany; 4 Institute for Biometrics and Epidemiology, German Diabetes Center, Leibniz Center for Diabetes Research at Heinrich-Heine-University, Düsseldorf, Germany; 5 Department of Ophthalmology, Medical Faculty and University Hospital Düsseldorf, Heinrich-Heine-University, Düsseldorf, Germany; 6 Department of Production, Hospital Pharmacy, Landesapotheke Salzburg, Salzburg, Austria; 7 Department of Neurology, Medical Faculty and University Hospital Düsseldorf, Heinrich-Heine-University, Düsseldorf, Germany; 8 Clinic for Orthopedics and Trauma Surgery, Medical Faculty and University Hospital Düsseldorf, Heinrich-Heine-University, Düsseldorf, Germany

**Keywords:** Antioxidative defence, ER stress, Hyperbaric oxygen therapy, Insulin resistance, Mitohormesis

## Abstract

**Aims/hypothesis:**

Hyperbaric oxygen (HBO) therapy may improve hyperglycaemia in humans with type 2 diabetes, but underlying mechanisms are unclear. Our objective was to examine the glucometabolic effects of HBO on whole-body glucose disposal in humans with type 2 diabetes.

**Methods:**

In a randomised placebo-controlled crossover trial located at the German Diabetes Center, 12 male individuals with type 2 diabetes (age 18–75 years, BMI <35 kg/m^2^, HbA_1c_ 42–75 mmol/mol [6–9%]), randomly allocated by one person, underwent 2-h HBO, once with 100% (240 kPa; HBO) and once with 21% oxygen (240 kPa; control, CON). Insulin sensitivity was assessed by hyperinsulinaemic–euglycaemic clamps with d-[6,6-^2^H_2_]glucose, hepatic and skeletal muscle energy metabolism were assessed by ^1^H/^31^P-magnetic resonance spectroscopy, while high-resolution respirometry measured skeletal muscle and white adipose tissue (WAT) mitochondrial capacity. All participants and people assessing the outcomes were blinded.

**Results:**

HBO decreased fasting blood glucose by 19% and increased whole-body, hepatic and WAT insulin sensitivity about one-third (*p*<0.05 vs CON). Upon HBO, hepatic γ-ATP concentrations doubled, mitochondrial respiratory control doubled in skeletal muscle and tripled in WAT (*p*<0.05 vs CON). HBO increased myocellular insulin-stimulated serine-473/threonine-308 phosphorylation of Akt but decreased basal inhibitory serine-1101 phosphorylation of IRS-1 and endoplasmic reticulum stress (*p*<0.05 vs CON).

**Conclusions/interpretation:**

HBO-mediated improvement of insulin sensitivity likely results from decreased endoplasmic reticulum stress and increased mitochondrial capacity, possibly leading to low-dose reactive oxygen species-mediated mitohormesis in humans with type 2 diabetes.

**Trial registration:**

ClinicalTrials.gov NCT04219215

**Funding:**

German Federal Ministry of Health, German Federal Ministry of Education and Research, North-Rhine Westfalia Ministry of Culture and Science, European-Regional-Development-Fund, German-Research-Foundation (DFG), Schmutzler Stiftung

**Graphical abstract:**

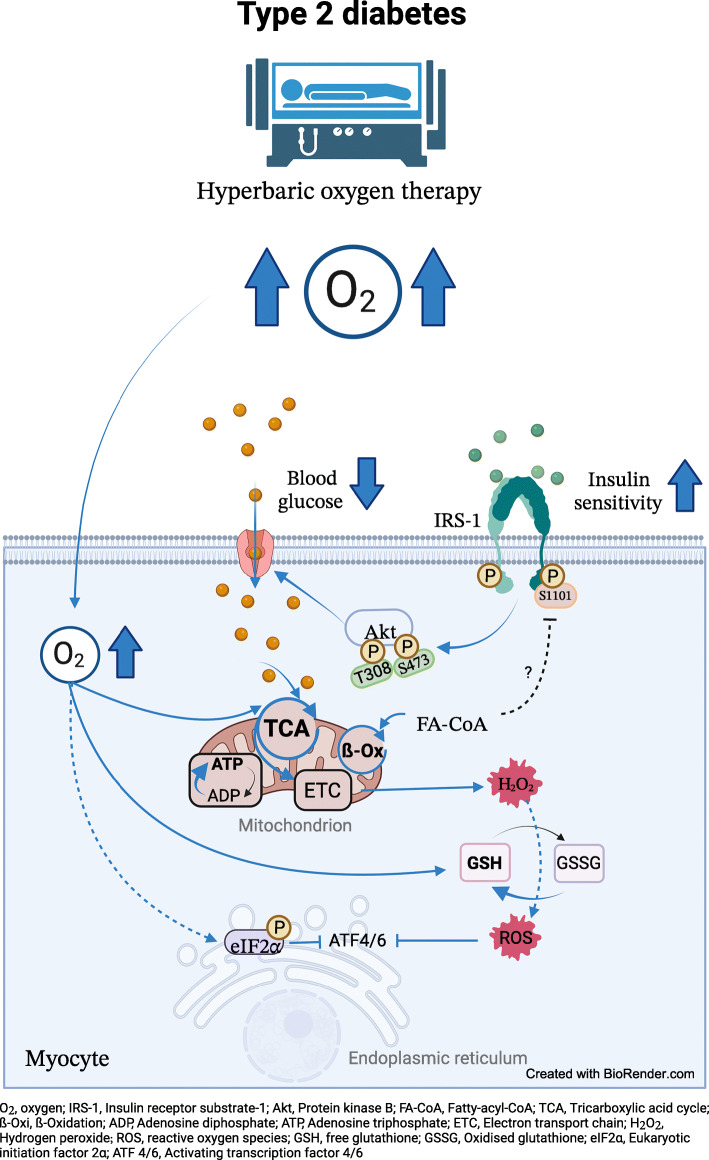

**Supplementary Information:**

The online version of this article (10.1007/s00125-022-05797-0) contains peer-reviewed but unedited supplementary material..

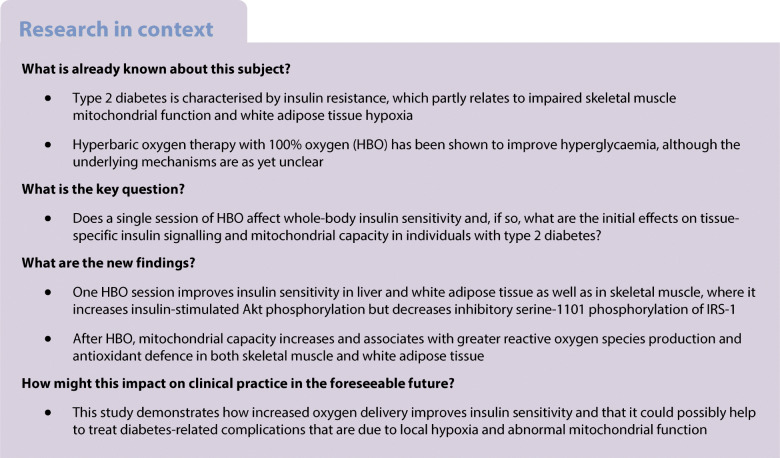



## Introduction

Various studies have suggested a causal relationship between oxidative stress and insulin resistance [1], an early hallmark of type 2 diabetes. Indeed, mechanisms involved in the development of insulin resistance comprise not only lipotoxicity and low-grade inflammation but also impaired muscle mitochondrial function [2]. In humans with obesity, whole-body (peripheral) insulin resistance was also found to be positively associated with white adipose tissue (WAT) hypoxia, inflammation and oxidative stress [2, 3], all of which represent initial abnormalities during the pathogenesis of type 2 diabetes [2]. However, according to the concept of mitohormesis, low-dose reactive oxygen species (ROS) not only cause oxidative stress but may rather function as signalling molecules inducing a protective response, including enhanced antioxidative capacity, against metabolic challenges [4].

Hyperbaric oxygen (HBO) treatment provides for 100% oxygen (O_2_) breathing during a rise of atmospheric pressure to 240 kPa for approximately 120 min. Depending on the medical indication (acute or chronic), the patients are exposed to 2–40 sessions in the HBO chamber. The higher ambient pressure during breathing of 100% O_2_ within the HBO chamber increases the partial pressure of O_2_ in the alveoli of the lung, resulting in 100% O_2_ saturation of haemoglobin and a fourfold increase in the amount of dissolved O_2_ in the blood [5]. The increased amounts of O_2_ physically dissolved in the blood leads to linear improvement of oxygenation of all tissues and improved mitochondrial metabolism, which is of clinical relevance for reaching injured or insufficient supplied tissues [6]. Interestingly, long-term HBO treatment of diabetic foot syndrome has been shown to acutely ameliorate hyperglycaemia [7]. It has been hypothesised that increased O_2_ delivery either directly stimulates metabolic fluxes or induces low-grade oxidative stress, which via mitohormesis may induce antioxidant defence and/or anti-inflammatory responses, as demonstrated in leukocytes [8]. In line, HBO therapy for four consecutive days increased whole-body insulin sensitivity in obese humans and altered systemic concentrations of certain inflammatory cytokines [8]. However, the underlying cellular mechanisms, specifically the initial effects on insulin signalling and mitochondrial capacity, have not yet been investigated, particularly in the context of type 2 diabetes.

**Fig. 1:**
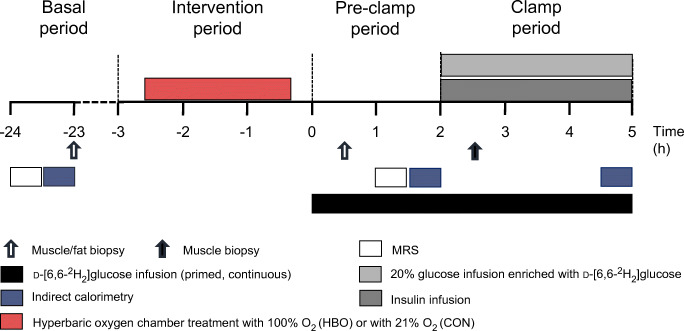
Study design. Participants with type 2 diabetes (*n*=12) randomly underwent two 2 h sessions in a hyperbaric chamber once with application of 100% O_2_ (at 240 kPa; HBO) and once with ambient air as control (21% O_2_; 240 kPa; CON) from −2.5 h to −0.5 h (intervention period) spaced by an interval of 3 weeks. Skeletal muscle and adipose tissue biopsies were taken at time points −23 h (basal period), +0.5 h (pre-clamp period) and +2.5 h (only skeletal muscle) during a hyperinsulinaemic–euglycaemic clamp test (clamp period), performed from +2 h to +5 h. In vivo ^1^H/^31^P-MRS was used to directly quantify hepatic and muscle lipid and energy metabolism

This study compared the acute effects of one single session of 100% O_2_ (HBO) with one of 21% O_2_ ambient air (control condition, CON), both delivered at 240 kPa compression, with regards to the following variables: (1) tissue-specific glucose metabolism during endogenous (fasting) insulinaemia; (2) tissue-specific insulin sensitivity during hyperinsulinaemic–euglycaemic clamp conditions; (3) skeletal muscle and WAT mitochondrial capacity, oxidative stress and antioxidant capacity; and (4) insulin signalling in skeletal muscle of humans with type 2 diabetes (Fig. 1 and ESM Table 1).

## Methods

### Volunteers

Fifteen male volunteers with type 2 diabetes were enrolled in this randomised, placebo-controlled, crossover clinical trial (ClinicalTrials.gov registration no. NCT04219215) but three volunteers were excluded prematurely. In total, 12 participants completed the study and data were analysed (ESM Table 1 and ESM Fig. 1). All participants were recruited from March 2018 to June 2019 according to the following inclusion criteria: type 2 diabetes (duration <7 years), age (18–75 years), BMI <35 kg/m^2^ and HbA_1c_ 42–75 mmol/ml (6–9%). Exclusion criteria included: uncontrolled hyperglycaemia (>13.3 mmol/l), diabetes types other than type 2 diabetes (ADA criteria), thiazolidinedione use during the preceding 6 months, clinically relevant angiopathy, restrictive or obstructive lung diseases, other acute or chronic diseases including wounds and the use of pharmacological agents known to affect insulin sensitivity, lipid metabolism or immunological function. Three days prior to all visits, participants were instructed to discontinue blood glucose-lowering medication and ingest a carbohydrate-rich diet. All participants gave their written informed consent before inclusion and underwent extensive screening tests. The study was performed according to the 2013 version of the Declaration of Helsinki and approved by the local institutional ethics board.

### Randomisation

The random allocation sequence (1:1) was generated by an experienced statistician at the German Diabetes Center (DDZ) (PB) using SAS software, version 9.3 (SAS Institute, Cary, NC, USA). Participants were randomly assigned to their treatment sequence by a person at the DDZ who was not involved in the conduct of the study. The randomisation list was kept by this person and was not accessible to the study personnel. Study participants, medical staff and researchers were blinded until completion of the study.

### Experimental protocol

The visits took place at an interval of 3 weeks. Each visit covered a period of 31 h, divided into four time periods (Fig. 1): basal (−24 h to −3 h); intervention (−3 h to +0 h); pre-clamp (+0 h to +2 h); and clamp (+2 h to +5 h). All participants arrived at the DDZ at 09:00 hours, after a 12 h overnight fast (−24 h to −3 h, basal period), for a 1 h session of MRI and magnetic resonance spectroscopy (MRS) followed by two biopsies from the abdominal subcutaneous WAT and vastus lateralis muscle as described before [9]. The participants were allowed to return home for the night and came back to the centre on the next day after another 12 h fast. At 06:00 hours (−3 h to 0 h, intervention period), two i.v. catheters were inserted into contralateral forearm veins. Then, participants received a 2 h HBO session according to the following protocol: the twin-lock, multi-place hyperbaric chamber (Sayers/ Hebold, updated by Haux, Germany, 2003) was compressed to 240 kPa under ambient air, followed by mask or hood breathing (100% O_2_ [HBO]/21% O_2_ [CON]) for 30 min three times each spaced by a 10 min break with breathing ambient air and a 30 min linear decompression to 101.3 kPa under 100% O_2_ (HBO)/21% O_2_ (CON), respectively, at the HBO Unit of the University Hospital Düsseldorf, Germany. Transcutaneous tissue oxygen (tcpO_2_) measurement served as diagnostic information for intervention performance before, during and after the intervention phase (ESM Table 2). From 0 h to +5 h during the pre-clamp and clamp periods, participants were given a continuous infusion (0.036 mg [kg body weight]^−1^ min^−1^) of d-[6,6-^2^H_2_]glucose (99% enrichment; Cambridge Isotope Laboratories, Andover, MA, USA), following a 10 min priming bolus (0.36 mg/[kg body weight]^−1^ min^−1^ [mg/dl fasting blood glucose]) [10]. At +0.5 h, the participants had another two biopsies from the subcutaneous WAT and skeletal muscle followed by another MRI/MRS session. From +2 h to +5 h (clamp period) they underwent a hyperinsulinaemic–euglycaemic clamp test (bolus 80 mU [m body surface area]^−2^ min^−1^ for 8 min, followed by continuous infusion of 40 mU [m body surface area]^−2^ min^−1^; human short-acting insulin [Insuman Rapid; Sanofi, Frankfurt, Germany]). Blood glucose concentration was maintained at 5 mmol/l by adapting the glucose infusion rate using 20% glucose (B. Braun AG, Melsungen, Germany) enriched with d-[6,6-^2^H_2_]glucose (ESM Table 3). Further blood samples were collected at pre-specified intervals. During the clamp period, a third skeletal muscle biopsy was obtained at +2.5 h.

### Indirect calorimetry

Indirect calorimetry was performed in the canopy mode with Vmax Encore 29n (CareFusion, Höchberg, Germany) during the last 30 min of the basal, pre-clamp and clamp periods [11].

### Skeletal muscle and WAT biopsy

Skeletal muscle and WAT biopsy samples were taken from the vastus lateralis muscle and subcutaneous adipose tissue of the lower abdomen, respectively, as described before [12].

### MRI and MRS

All MRI/MRS measurements were conducted on a 3.0-T MR scanner (Achieva X-series; Philips Healthcare, Best, the Netherlands). Intrahepatic lipid (IHL) and intramyocellular lipid (IMCL) contents were quantified by ^1^H-MRS. Hepatic γ-ATP and total inorganic phosphate (P_i_) contents were measured with ^31^P-MRS using a 14 cm circular surface coil, as reported before [11]. All acquired spectra were processed using jMRUI software, version 5.2 (http://nmr.isibrno.cz/jmrui.html). Absolute concentrations of IHL, ATP and P_i_ were calculated as reported [11, 13]. The concentration of IMCL was calculated from the peak areas of -CH_2_ (methylene) peaks at 1.3 ppm and 1.5 ppm with respect to the water peak area and data were corrected for T_1_ and T_2_ relaxation effects derived from a previous study [14], calculated as the CH_2_/water ratio and expressed as a percentage.

### High-resolution respirometry

Ex vivo analysis of mitochondrial respiration was performed in permeabilised muscle fibres and WAT using high-resolution respirometry (Oxygraph-2k; Oroboros, Innsbruck, Austria), as previously described [15]. Maximal mitochondrial respiration with electron input through CI and CII was determined after addition of malate (2 mmol/l), pyruvate (10 mmol/l) and glutamate (10 mmol/l), followed by ADP (2.5 mmol/l) and succinate (10 mmol/l); cytochrome C (10 μmol/l) was added to test the integrity of the outer mitochondrial membrane. Oligomycin (5 μmol/l), an inhibitor of ATP synthase, was added to determine oligomycin-induced leak respiration. Finally, electron transport system capacity (i.e. uncoupled mitochondrial respiration) was assessed by titration with carbonyl cyanide *p*-(trifluoromethoxy)-phenylhydrazone (FCCP). Antimycin A (5 μmol/l) was added to assess non-mitochondrial-driven respiration by inhibiting CIII. Hydrogen peroxide production in permeabilised muscle fibres was quantified by high-resolution respirometry with Amplex Red, as previously described [15]. Citrate synthase activity (CSA) was measured spectrophotometrically (Sigma-Aldrich, St Louis, MO, USA) according to Morgunov and Srere [16] and normalised to protein concentration (Bicinchoninic acid assay kit, Sigma-Aldrich).

### Analyses of lipid peroxidation, antioxidative capacity and oxidative stress

Concentrations of thiobarbituric acid reactive substances (TBARS) were assessed fluorometrically in serum and biopsy samples from subcutaneous fat and skeletal muscle according to the manufacturer’s instructions (BioTek, Bad Friedrichshall, Germany) [17].

In skeletal muscle and WAT samples, total glutathione and oxidised glutathione (GSSG) contents were quantified colorimetrically and normalised to protein concentration (Thermo Fisher Scientific, Dreieich, Germany), based on the method of Griffith [18]. Free glutathione (GSH) concentrations were calculated by subtracting GSSG from the total glutathione content, as a marker of tissue antioxidative capacity [19]. Static oxidation reduction potential (sORP; in mV) and capacity oxidation reduction potential (cORP; in μC), as markers of systemic oxidative stress, were determined in 20 μl serum samples (RedoxSYS instrument; Luoxis Diagnostics, Englewood, CO, USA) [20].

### GC-MS

Determination of atom per cent enrichment of blood [^2^H_2_]glucose was performed on a Hewlett Packard 6890 gas chromatograph equipped with a 25 m CPSil5CB capillary column (0.2 mm i. d., 0.12 μm film thickness; Chrompack/Varian, Middelburg, the Netherlands) and interfaced to a Hewlett Packard 5975 mass selective detector [11].

### Western blotting

Expression levels of proteins of interest were assessed by western blot analysis, as described before [15]. Data were normalised to housekeeping protein or total protein and expressed in arbitrary units. The following primary antibodies were purchased from Cell Signaling Technology and diluted 1:1000, unless differently specified: binding immunoglobulin protein (BiP) (3177); activating transcription factor (ATF) 6 (65880); eukaryotic initiation factor 2α (eIF2α) (9722); serine-51 phosphorylation of eIF2α (3398); serine-1101 phosphorylation of IRS-1 (2385); serine-307 phosphorylation of IRS-1 (2381); Akt (9272); serine-473 phosphorylation of Akt (9271); threonine-308 phosphorylation of Akt (9275); and GAPDH (1:5000) (2118) as housekeeping. The IRS-1 antibody (06-248; purchased from Millipore, Burlington, MA, USA) and ATF4 antibody (10835-1-AP; Proteintech, Rosemont, IL, USA) were diluted 1:1000 in 5% milk in TBST. After incubation overnight with the primary antibodies, the membranes were washed and incubated with the respective horseradish peroxidase (HRP)–conjugated secondary anti-rabbit (7074, CST) or anti-mouse (7076, CST) antibodies, diluted 1:2500 and 1:1000 respectively, in 5% milk in TBST.

### Measurement of circulating metabolites and hormones

Plasma concentrations of insulin, glycerol, NEFA, alanine aminotransferase, aspartate aminotransferase, triacylglycerol and chylomicrons, as well as whole-blood measurements of glucose and HbA_1c_, were analysed as previously described [11]. Serum concentrations of IL-6, IL-1 receptor antagonist (IL-1ra), TNF-α, fibroblast growth factor-21 (FGF-21), myeloperoxidase (MPO) were quantified by Quantikine HS (IL-6, TNF-α) or Quantikine (IL-1ra, FGF-21, MPO) ELISA kits (R&D Systems/BioTechne, Wiesbaden, Germany) and superoxide dismutase 3 (SOD3) by ELISA (Cloud-Clone, Katy, TX, USA), as described [21]. Serum concentrations of total and high-molecular-weight adiponectin were measured by ELISA (ALPCO, Salem, NH, USA) [11].

### Calculations

During pre-clamp and clamp periods, whole-body glucose disposal rate (*R*_d_) was calculated from [^2^H_2_]glucose enrichments (Steele’s steady-state equation). During the pre-clamp period, hepatic ATP/P_i_ was calculated as a reliable index of the hepatic cytosolic energy status and/or phosphorylation potential [22]. Hepatic insulin clearance was calculated as the ratio of fasting plasma C-peptide and fasting plasma insulin concentrations during the pre-clamp period (0 h to +2 h) [23]. During the last 30 min of the pre-clamp period (+1.5 h to +2 h), fasting endogenous glucose production (EGP) and fasting NEFA were multiplied by fasting insulin levels to reflect hepatic glucose production and adipose tissue insulin resistance, respectively [11]. During the steady-state clamp period (+4.5 h to +5 h), EGP and NEFA suppression were calculated to assess hepatic and WAT insulin sensitivity, respectively [11]. From indirect calorimetry, whole-body resting energy expenditure (REE) and respiratory exchange ratio (RER) were calculated from measurements of respiratory $\dot{V}{\mathrm{O}}_2$ and carbon dioxide production by the Weir equation [24, 25]. Glucose oxidation (GOX), lipid oxidation (LOX) and non-oxidative glucose disposal (NOXGD) were calculated according to Frayn [26]. Deltas were calculated as difference (Δ) in the respective variable between pre-clamp and basal periods. Incremental AUCs (iAUCs) were calculated (pre-clamp and clamp period combined) using the trapezoidal rule, corrected for the respective AUC.

### Statistics

The power calculation was based on a previous study on HBO-induced glucose lowering, using two simultaneous two-sided paired *t* tests resulting in a sample size of *n*=10 with a multiplicity-adjusted *α* of 0.025 and a power of 85% [8]. Results are presented as means±SEM (in figures), means±SD for normally distributed data, or median with IQR (first to third quartile) for log normally distributed variables (in ESM tables), and compared by mixed-model repeated-measures ANOVA (mixed-model ANOVA) adjusted for BMI and age, and with Tukey–Kramer correction. Comparison of changes within one time point was done using a crossover test. Variables with skewed distributions were log_*e*_-transformed before analysis. Statistical significance of differences was defined at *p*<0.05. Calculations were performed using SAS version 9.4 (SAS Institute, Cary, NC, USA).

## Results

### HBO raises tissue oxygen pressure

During HBO treatment, tissue oxygen pressure as measured by tcpO_2_ increased 13-fold compared with baseline and was 6.5-fold higher than during CON (*p*<0.001, ESM Table 2). After HBO, tissue oxygen pressure continued to be 2.7-fold increased above baseline values and was 2.5 times higher than in CON (*p*<0.05, ESM Table 2). Blood oxygen saturation was about 98% throughout the studies (*p*<0.999, ESM Table 2).

### HBO rapidly improves hyperglycaemia

**Fig. 2:**
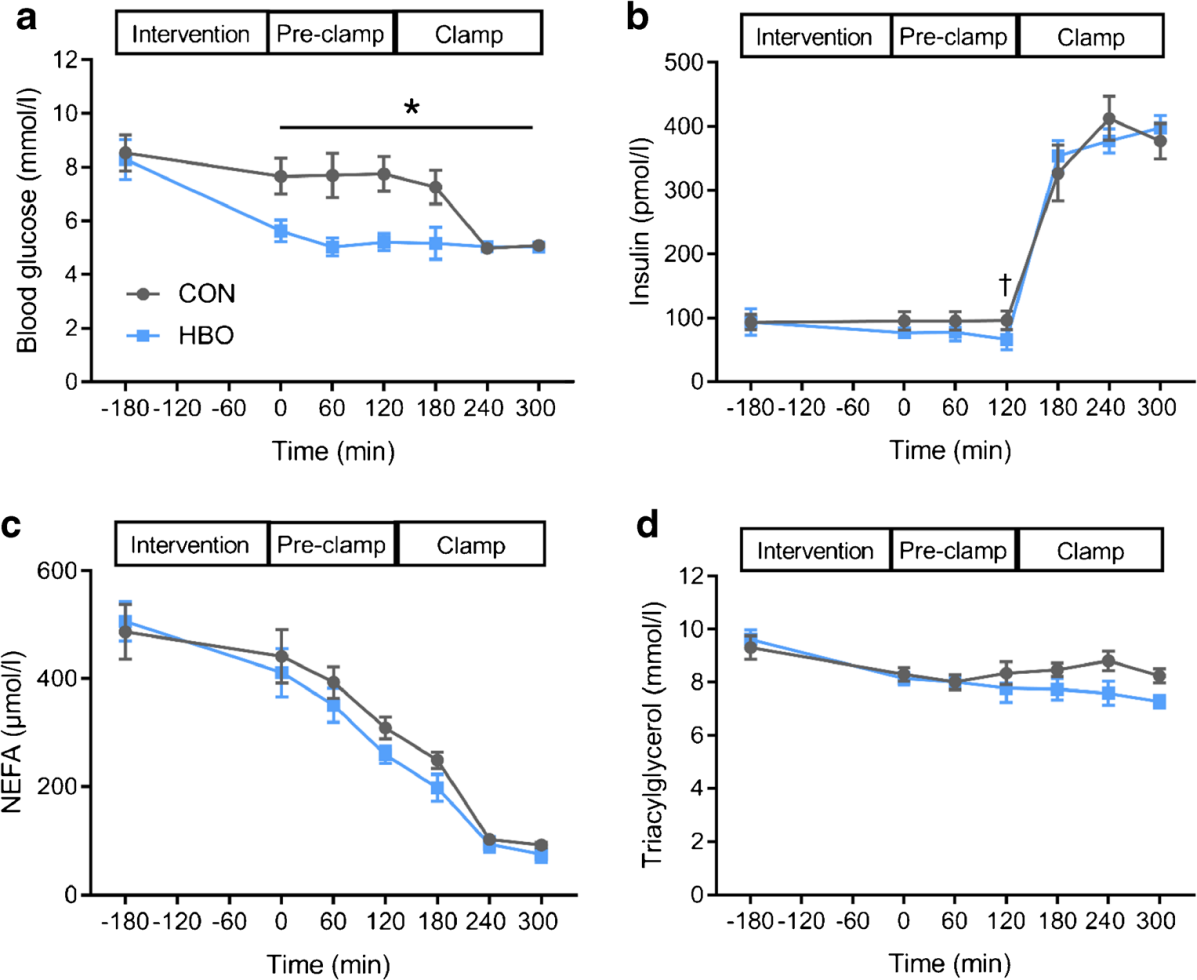
Time course of circulating metabolites and hormones in humans with type 2 diabetes before and after HBO or CON treatment. Concentrations (means ± SEM) of fasting blood glucose (**a**), plasma insulin (**b**), plasma NEFA (**c**) and plasma triacylglycerol (**d**) in humans with type 2 diabetes (*n*=12, except for plasma triacylglycerol *n*=10) after two 2 h sessions in a hyperbaric chamber with either 100% O_2_ (HBO) or 21% O_2_ ambient air (CON) from −2.5 h to −0.5 h (intervention period). *p<0.05 vs CON for iAUC calculated for pre-clamp and clamp periods combined using the trapezoidal rule corrected for the respective AUC; ^†^*p*<0.05 for HBO vs CON at time point +120 min, applying the crossover test

After HBO treatment, blood glucose decreased by 19% from baseline compared with CON (iAUC *p*=0.003; Fig. 2a). Plasma insulin concentration was 35% lower after HBO than after CON at time point +120 min (*p*=0.031) but did not differ during the pre-clamp and clamp period (iAUC *p*=0.880, Fig. 2b). C-peptide (iAUC 12.2±2.2 HBO and 14.1±2.1 CON, *p*=0.784; ESM Fig. 2), NEFA (iAUC, *p*=0.828, Fig. 2c) and triacylglycerol (iAUC, *p*=0.181, Fig. 2d) did not differ between HBO and CON. In addition, hepatic insulin clearance was not different between the interventions (6.51±1.12 HBO and 6.87±1.86 CON, *p*=0.592).

### HBO leads to improvement of hepatic, skeletal muscle and WAT insulin sensitivity

**Fig. 3:**
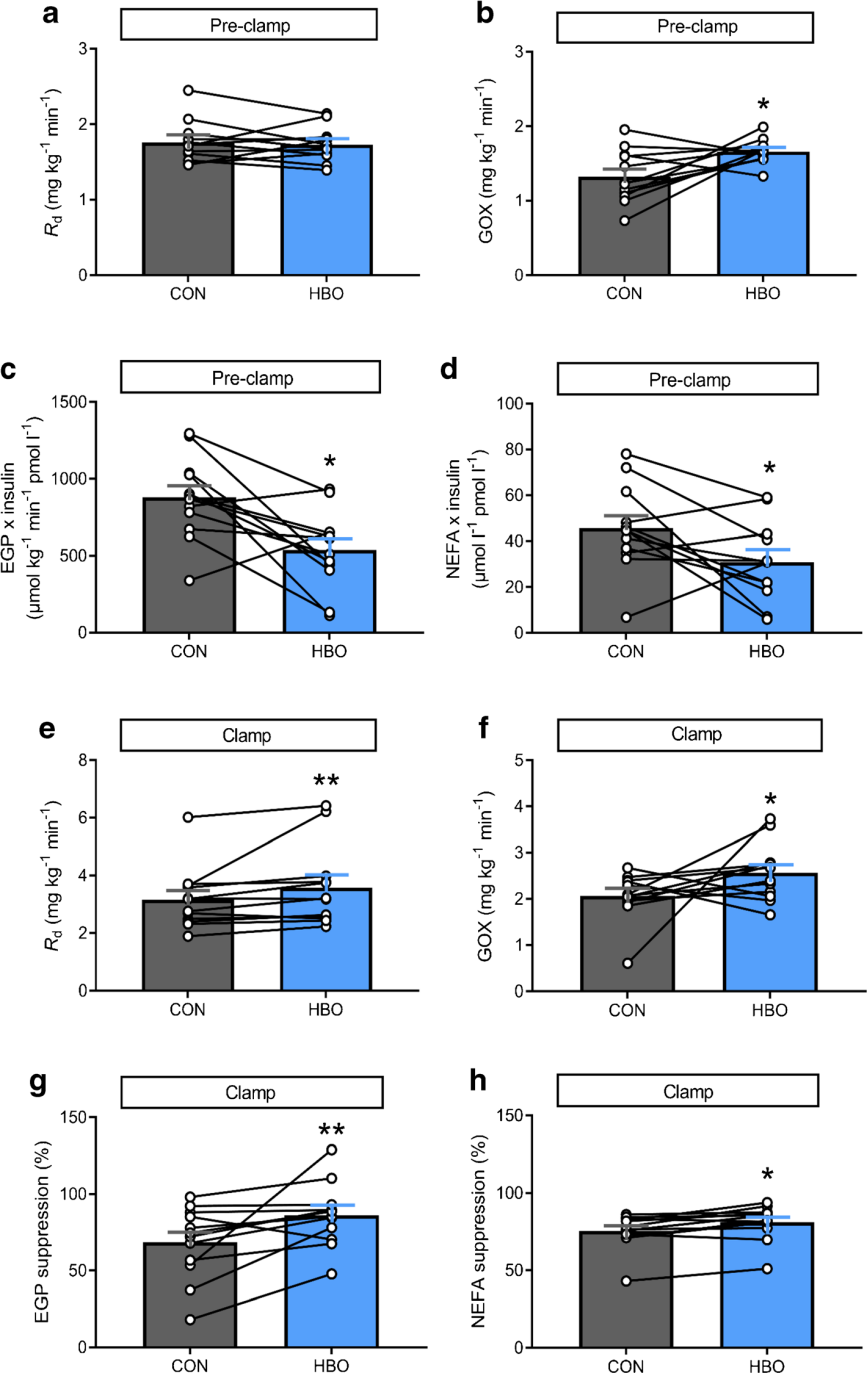
Whole-body and tissue-specific glucose and lipid metabolism after HBO or CON treatment in humans with type 2 diabetes. (**a**–**d**) Pre-clamp period (0 h to +2 h) *R*_d_ (**a**), GOX (**b**), insulin-adjusted EGP (EGP × insulin; **c**) and adipose tissue insulin resistance from circulating NEFA (plasma NEFA × insulin; **d**). (**e**–**h**) Clamp period (+2 h to +5 h) insulin-stimulated *R*_d_ (**e**), GOX (**f**), per cent EGP suppression (**g**) and per cent NEFA suppression (**h**). Data are presented as means ± SEM (*n*=12) for humans with type 2 diabetes after two 2 h sessions in a hyperbaric chamber with either 100% O_2_ (HBO) or 21% O_2_ ambient air (CON) from −2.5 h to −0.5 h (intervention period). *p<0.05 and **p<0.01 vs CON (crossover test)

During the pre-clamp period, REE (*p*=0.15, ESM Table 4) and RER (*p*=0.67, ESM Table 4), as well as *R*_d_ (*p*=0.59; Fig. 3a), were not different between interventions, while GOX was increased by about 25% after HBO vs CON treatment (*p*=0.028, Fig. 3b and ESM Table 4). Rates of LOX (*p*=0.025; ESM Table 4), and liver (*p*=0.024; Fig. 3c) and WAT insulin resistance (*p*=0.042; Fig. 3d), were all about one-third lower after HBO than after CON. During the clamp period, REE and RER increased after HBO (*p*=0.021 and *p*=0.039, respectively) and CON (*p*=0.037 and *p*=0.035, respectively), compared with the basal period, but without differences between the interventions (*p*=0.11 and *p*=0.64, respectively; ESM Table 4). During the clamp period, LOX tended to be lower after HBO compared with CON treatment but the difference did not reach statistical significance (*p*=0.081; ESM Table 4). However, whole-body insulin sensitivity was 11% higher after HBO than after CON (*p*=0.005; Fig. 3e), mainly due to 20% higher GOX (*p*=0.008; Fig. 3f) and not to NOXGD (*p*=0.264). In addition, hepatic and WAT insulin sensitivity were 20% (*p*=0.008) and 8% (*p*=0.039) higher upon HBO compared with CON during the clamp period (Fig. 3g,h).

### HBO acutely stimulates energy metabolism but also increases oxidative stress and antioxidative defence

**Fig. 4:**
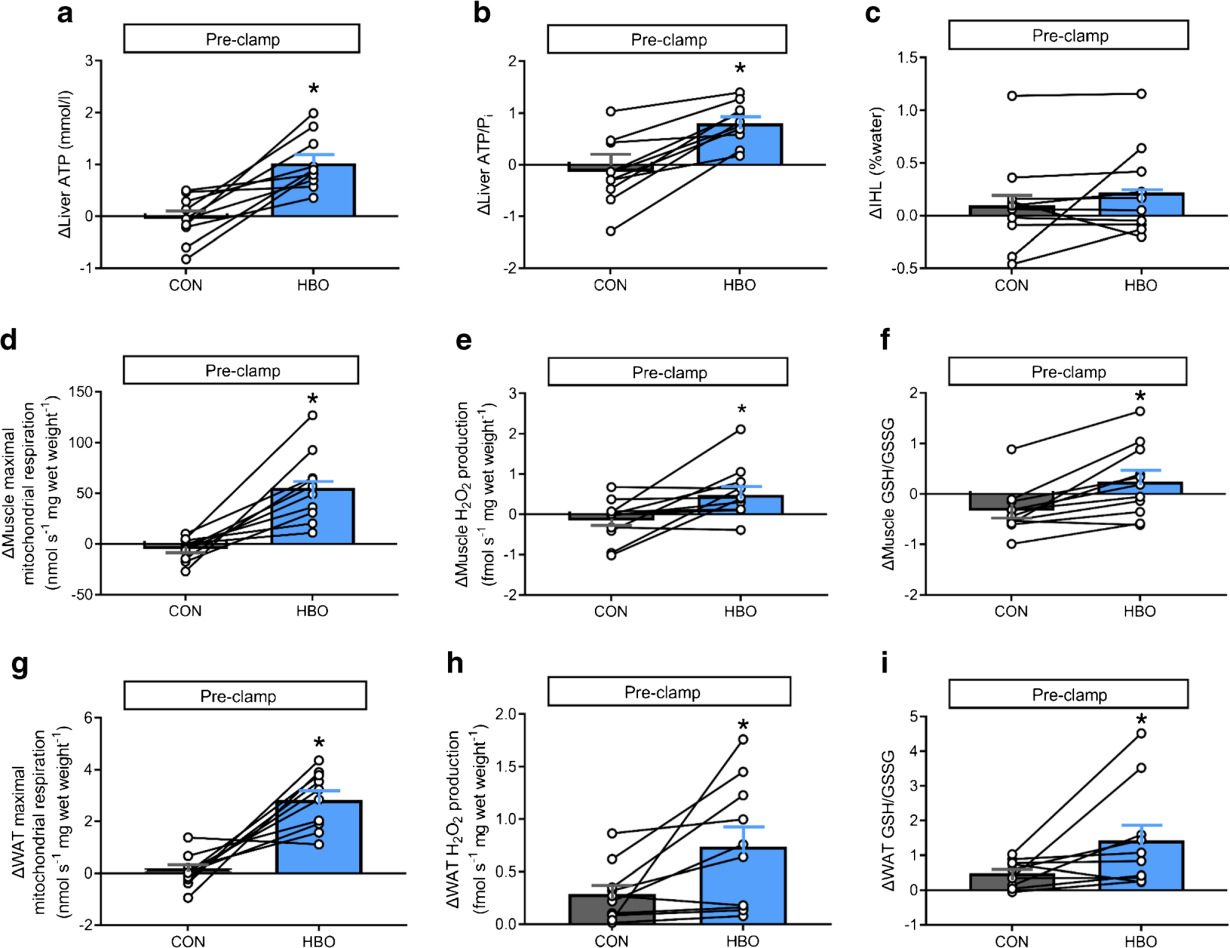
Variables of tissue-specific energy metabolism and antioxidative capacity after HBO or CON treatment in humans with type 2 diabetes. Difference (Δ) between pre-clamp and basal periods for hepatic ATP concentration (**a**), hepatic ATP/P_i_ concentration (**b**), IHL concentration (**c**), skeletal muscle maximal mitochondrial respiration with electron input through CI + CII combined (**d**) and skeletal muscle maximum mitochondrial production of H_2_O_2_ (**e**), as well as the ratio of reduced/oxidised glutathione (GSH/GSSG) (**f**), WAT maximal mitochondrial respiration with electron input through CI + CII combined (**g**), WAT maximum mitochondrial production of H_2_O_2_ (**h**) and GSH/GSSG level (**i**). Data are presented as means ± SEM for humans with type 2 diabetes (*n*=10, except for hepatic ATP/P_i_ and IHL concentration *n*=12) after two 2 h sessions in a hyperbaric chamber with either 100% O_2_ (HBO) or 21% O_2_ ambient air (CON) from −2.5 h to −0.5 h (intervention period). *p<0.05 vs CON (crossover test)

Changes were analysed by comparing the difference (Δ) between pre-clamp and basal periods for each intervention. Non-invasive ^31^P-MRS revealed that hepatic ATP concentrations rose markedly by 101% after HBO compared with CON (ΔATP, *p*=0.031, Fig. 4a and ESM Table 5); hepatic concentrations of P_i_ were comparable between HBO and CON (ΔP_i_, *p*=0.999; ESM Table 5). Hepatic ATP/P_i_ ratio rose by 89% after HBO compared with CON (Δliver ATP/P_i_, *p*=0.031, Fig. 4b), whereas IHL (ΔIHL, *p*=0.57, Fig. 4c and ESM Table 5) and IMCL (ΔIMCL, *p*=0.99, ESM Table 5) did not differ.

Changes in skeletal muscle mitochondrial CSA were comparable between HBO and CON (ΔCSA, 5.9±1.1 and 3.5±1.3 CON [nmol min^−1^ (mg protein)^−1^], *p*=0.551). Upon HBO, the change in skeletal muscle maximal mitochondrial respiration with electron input through CI + CII was 1200% higher compared with CON (ΔCI + CII, *p*=0.015, Fig. 4d). Change in mitochondrial respiration control ratio (ΔRCR, 0.89±0.42 vs 0.42±0.12 [state 3/ state o], *p*=0.031) doubled, while change in leak control ratio was not different (ΔLCR, 0.27±0.09 vs 0.03±0.09 [state o/ state u], *p*=0.903) compared with CON. After HBO, the change in muscle H_2_O_2_ was 163% greater (Δmuscle H_2_O_2_, *p*=0.012, Fig. 4e) and the change in myocellular levels of TBARS was gradually higher (ΔTBARS, 34±22 vs CON −24±16 [pmol (mg protein)^−1^], *p*=0.062). In parallel, the change in muscle GSH/GSSG ratio was 68% higher after HBO compared with CON (Δmuscle GSH/GSSG, *p*=0.008, Fig. 4f).

In WAT, the change in CSA did not differ between the interventions (ΔCSA, 4.8±1.6 HBO vs 3.1±1.4 CON [nmol min^−1^ (mg protein)^−1^], *p*>0.999). Changes in WAT CI + CII (ΔCI + CII, 280%, *p*=0.008 vs CON, Fig. 4g) and RCR (ΔRCR, 3.3±0.5 vs −1.0±0.6 [state 3/ state o], *p*=0.031) were markedly higher upon HBO than CON. Change in LCR was not different between interventions (ΔLCR, −1.39±0.68 vs −2.93±3.99 [state o/ state u], *p*=0.717). HBO further raised H_2_O_2_ production (ΔWAT H_2_O_2_, 160%, *p*=0.009, Fig. 4h), TBARS (ΔTBARS, 374±140 vs −56±68 [pmol (mg protein)^−1^], *p*=0.0020) and GSH/GSSG ratio compared with CON (ΔWAT GSH/GSSG, 186%, *p*=0.031, Fig. 4i). Of note, all measured serum biomarkers of systemic oxidative stress (i.e. TBARS, sORP and cORP) were not different when comparing interventions (iAUCs *p*>0.05; ESM Table 6).

### HBO acutely improves myocellular insulin signalling while reducing endoplasmic reticulum stress

**Fig. 5:**
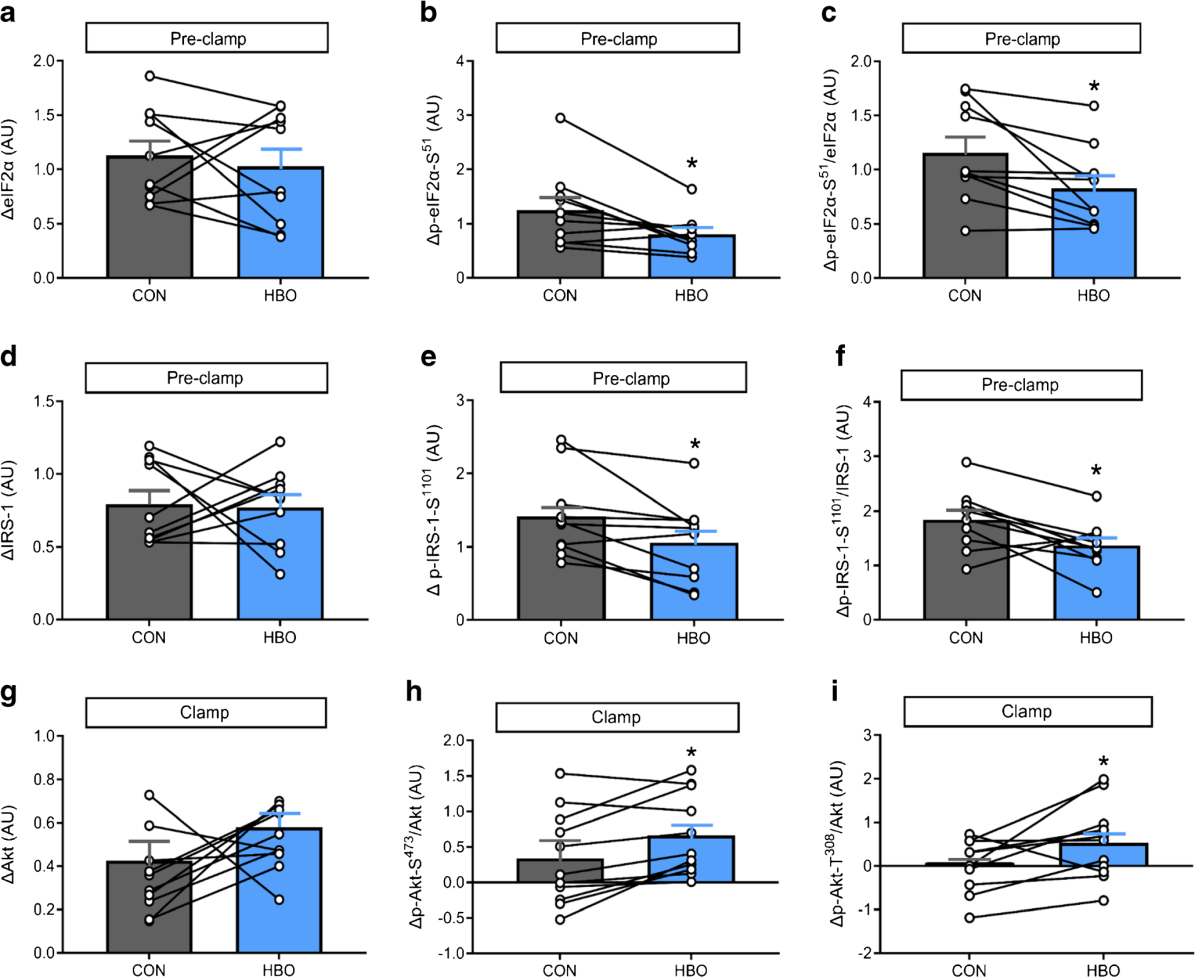
Myocellular ER stress and insulin signalling after HBO or CON in humans with type 2 diabetes. (**a**–**f**) Difference (Δ) between pre-clamp and basal periods for eIF2α (**a**), serine-51 phosphorylation of eIF2α (p-eIF2α-S^51^; **b**) and p-eIF2α-S^51^/eIF2α ratio (**c**), as well as IRS-1 (**d**), serine-1101 phosphorylation of IRS-1 (p-IRS-1-S^1101^; **e**) and Δp-IRS-1-S^1101^/IRS-1 ratio (**f**). (**g**–**i**) Difference (Δ) between clamp and basal periods for Akt (**g**), serine-473 phosphorylation of Akt per Akt (p-Akt-S^473^/Akt; **h**) and threonine-308 phosphorylation of Akt per Akt (p-Akt-T^308^/Akt; **i**). Data are presented as means ± SEM for humans with type 2 diabetes (*n*=10) after two 2 h sessions in a hyperbaric chamber with either 100% O_2_ (HBO) or 21% O_2_ ambient air (CON) from −2.5 h to −0.5 h (intervention period). *p<0.05 vs CON (crossover test). AU, arbitrary unit

First, changes were analysed by comparing the difference (Δ) between pre-clamp and basal periods. During the pre-clamp period, expression of biomarkers of endoplasmic reticulum (ER) stress was measured in skeletal muscle. Expression levels of eIF2α were comparable between the interventions (ΔeIF2α, *p*=0.69, Fig. 5a), while serine-51 phosphorylation of eIF2α and the phosphorylated eIF2α-S^51^/eIF2α ratio decreased by 35% and 28%, respectively, upon HBO (Δp-eIF2α-S^51^, *p*=0.019 and Δp-eIF2α-S^51^/eIF2α, *p*=0.006 vs CON, Fig. 5b, c). ATF4, ATF6 and BiP were 51%, 37% and 30% lower, respectively, after HBO (ΔATF4, *p*=0.027; ΔATF6, *p*=0.039; ΔBiP, *p*=0.037 all vs CON; ESM Table 7). Skeletal muscle IRS-1 protein expression levels did not differ between both interventions (ΔIRS-1, *p*=0.810, Fig. 5d). After HBO, however, inhibitory serine-1101 phosphorylation of IRS-1 and serine-1101 phosphorylation of IRS-1 per IRS-1 were 25% and 28% lower, respectively, compared with CON (Δp-IRS-1-S^1101^, *p*=0.019; Δp-IRS-1-S^1101^/IRS-1, *p*=0.046; Fig. 5e, f and ESM Table 8). Serine-307 phosphorylation of IRS-1 and serine-307 phosphorylation of IRS-1 per IRS-1 did not differ between interventions (Δp-IRS-1-S^307^, *p*=0.57; Δp-IRS-1-S^307^/IRS-1, *p*=0.43; ESM Table 8). Skeletal muscle Akt (*p*=0.87, ESM Table 8), serine-473 phosphorylation of Akt per Akt (p-Akt-S^473^/Akt, *p*=0.71; ESM Table 8) and threonine-308 phosphorylation of Akt per Akt (p-Akt-T^308^/Akt, *p*=0.83; ESM Table 8) protein expression levels were not different between the interventions during the pre-clamp period.

For the clamp period, changes were analysed by comparing the difference (Δ) between clamp and basal periods. Skeletal muscle Akt was not different between the groups (ΔAkt, *p*=0.101, Fig. 5g and ESM Table 8). After HBO, however, activating serine-473 and threonine-308 phosphorylation of Akt per Akt were 48% and 97% higher, respectively, compared with CON (Δp-Akt-S^473^/Akt, *p*=0.037; Δp-Akt-T^308^/Akt, *p*=0.024; Fig. 5h,i and ESM Table 8).

### HBO does not immediately affect biomarkers of systemic low-grade inflammation

Serum IL-6, IL-1ra, TNF-α, FGF-21, MPO, SOD3, and total and high-molecular-weight adiponectin were not different between interventions (iAUCs *p*>0.05, ESM Table 9).

## Discussion

This study demonstrates that a single HBO treatment with 100% O_2_ is sufficient to improve insulin sensitivity rapidly and simultaneously in skeletal muscle, liver and WAT. In skeletal muscle, the improvement in insulin sensitivity was associated with increased myocellular insulin-stimulated Akt phosphorylation but also with reduced basal inhibitory serine-1101 phosphorylation of IRS-1. Further, marked improvements in mitochondrial capacity were paralleled by stimulation of ROS and antioxidative defence in both skeletal muscle and WAT. In the absence of systemic low-grade inflammation, these data suggest a role for cellular mitohormesis in HBO-induced improvement of insulin sensitivity.

The observation of a clinically meaningful reduction in fasting hyperglycaemia in humans with type 2 diabetes extends previous data [8, 27] insofar as a single session of HBO suffices for a glucose-lowering effect lasting up to 5.5 h after leaving the HBO chamber. This study confirmed the increase in insulin-stimulated whole-body glucose disposal reported for HBO previously [8, 27] but further detected an early improvement in fasting glucose metabolism in the liver and lipolysis in WAT by employing stable isotope dilution technique in humans with type 2 diabetes.

The novelty resides in the investigation of the initial tissue-specific cellular mechanisms underlying the beneficial metabolic effects of HBO treatment. In general, improvements in tissue-specific insulin sensitivity in type 2 diabetes may result from reductions in lipotoxic insulin signalling, low-grade inflammation, and oxidative and/or ER stress [2, 28]. Relative hypoxia in WAT [29] and skeletal muscle [30] has been associated with insulin resistance in obese humans with and without type 2 diabetes; thus hyperoxygenation may be expected to partially reverse insulin resistance. Indeed, the present study supports and extends such a concept, in that a single HBO session increased mitochondrial oxidative capacity in both WAT and skeletal muscle as well as hepatic ATP concentrations. Non-invasive quantification by ^31^P-MRS of the absolute concentrations of hepatic ATP and P_i_ is an established measure of hepatic energy metabolism and tightly correlates with hepatic flux through ATP synthase [31]. The observed rapid rise in hepatic ATP levels, as well as ATP/Pi ratios, by HBO is of particular interest, as humans with type 2 diabetes are known to exhibit about 25% lower hepatic energy metabolism than glucose-tolerant humans of similar age and body mass [31, 32]. Furthermore, the present study showed a greater improvement of insulin sensitivity in the liver when compared with whole-body (muscle) and WAT, suggesting an important role for hepatic energy metabolism in the glucose-lowering effect of HBO. Indeed, hepatic ATP synthesis correlates positively with short- and long-term glycaemic control, indicating that defective hepatic energy metabolism may reflect poor glucometabolic control in type 2 diabetes [32]. Mitochondria orchestrate hepatocellular energy metabolism via ATP synthesis and fatty acid oxidation [33]. In obesity, type 2 diabetes and non-alcoholic fatty liver disease, mitochondrial capacity can at least transiently be upregulated in response to excessive fuel availability, thereby preventing lipid deposition and modulate insulin resistance [2, 33]. In line, enhancing hepatic mitochondrial long-chain fatty acid oxidation capacity reversed hepatic insulin resistance and steatosis in obese mouse models [32]. However, whether the observed rise in hepatocellular ATP levels results from higher cellular oxygen availability or alternatively from enhanced mitochondrial substrate usage and thus improvement of insulin sensitivity and ectopic lipid deposition remains to be elucidated.

We also found that short-term HBO leads to higher production of ROS as well as antioxidative defence markers in both skeletal muscle and WAT of humans with type 2 diabetes. While chronic ROS overproduction can cause mitochondrial damage and reduced function in type 2 diabetes [34], short-term HBO treatment of diabetic rodents also increased whole-body insulin sensitivity despite elevated mitochondrial ROS production along with increased mRNA expression of biomarkers of antioxidative defence [35]. In addition, longer-term HBO treatment simultaneously stimulated ROS production and antioxidative capacity and in turn ameliorated chronic foot ulcers in humans with type 2 diabetes [36]. These data suggest operation of mitohormesis, which describes a non-linear hormetic response of mitochondrial ROS production to potentially harmful stressors like short-term hyperoxygenation, with low doses of ROS having less and high doses more toxic effects [4]. In line, low-dose ROS cellular signalling has been associated with higher expression levels of antioxidative enzymes [4]. Further, ROS can induce activation of IRS-1 via the ‘redox paradox’ [37]. This might also help to explain the present observation of acute reduction in the myocellular inhibitory serine-1101 phosphorylation of IRS-1, generally known to mediate lipid-induced or chronic insulin resistance in human skeletal muscle [2, 38]. Alternatively, the marked enhancement of WAT and muscle oxidative capacity by HBO could have reduced serine phosphorylation of IRS-1 and in turn stimulated distal insulin signalling pathways (Akt) via lower accumulation of lipotoxins such as diacylglycerols or ceramides [2, 38]. However, long-term HBO is also known to improve phosphorylated Akt levels through decreased IL-1β activity, at least in chondrocytes with insulin/IGF-1-induced chondrogenesis [39]. So far, enhanced Akt phosphorylation has been reported after short-term treatment with HBO only in high-fat diet mouse models [40] but not in humans.

This study also provides evidence for a substantial decrease in biomarkers of ER stress after a single session of HBO in humans with type 2 diabetes. ER stress has been shown to be involved in the pathogenesis of type 2 diabetes by leading to pancreatic beta cell loss and insulin resistance [41]; ER stress is also activated by acute diseases such as pancreatitis [42]. A previous study investigating hypoxia-altered cell signalling in endothelial cell culture demonstrated an acute increase in ER stress markers within hours [43]. Recently, moderate mitochondrial stress has been associated with ROS-dependent reduction of ER stress promoting metabolic health in rodents [44]. Thus, one may therefore speculate that HBO-mediated interaction of mitochondrial and ER stress could improve overall cellular performance thereby contributing to the improved insulin sensitivity.

Interestingly, the present study failed to detect any changes in circulating biomarkers of subclinical inflammation in humans with type 2 diabetes after HBO. While this is in line with a previous study [45], other studies report divergent results (e.g. reduced monocyte chemoattractant protein-1 and TNF-α after four HBO therapy sessions [8], or elevated IL-6, TNF-α and endothelin-1) [46]. Thus, short-term hyperoxygenation improves whole-body insulin sensitivity primarily via cellular mechanisms such as oxidative and ER stress, while long-term hyperoxygenation could act via amelioration of WAT inflammation, reduction of proinflammatory cytokine release [2] and/or alteration of cellular inflammatory pathways, as indicated by human studies on the effects of HBO on wound healing [36].

The limitations of this study include the use of a single dose of HBO, which does not allow extrapolation to long-term metabolic effects. Second, the applied study design neither clarifies how long the glucose-lowering effect persists after one session of HBO nor allows for detailed insights into changes in lipid metabolism upon HBO. Third, the study comprised male volunteers with type 2 diabetes, so findings cannot be generalised to women or to people with normoglycaemia or impaired glucose tolerance. In addition, the results may not be generalisable to all individuals with type 2 diabetes because of the short known disease duration as well as the rather narrow range of age and of insulin resistance, given the different diabetes endotypes [47], in the present study population. Furthermore, the absence of a glucose-tolerant group limits conclusions regarding the general population. Finally, studies on the hepatic molecular mechanisms were limited due to ethical reasons, impeding liver biopsies for this purpose. Nevertheless, absolute hepatic ATP concentrations (as measured in vivo with ^31^P-MRS) have been shown to correlate with in vivo flux through hepatic ATP synthase [9, 32]. Moreover, the present study measured hepatic ATP concentrations twice within one experiment so that the difference in ATP over time may serve as a surrogate of net ATP synthesis [31].

In conclusion, one single treatment of HBO with 100% O_2_ achieved the following outcomes: (1) rapid improvement in insulin sensitivity in skeletal muscle, liver and WAT; (2) marked improvement in mitochondrial functionality in all these tissues; (3) parallel stimulation of both ROS and antioxidative defence in skeletal muscle and WAT; (4) improved myocellular insulin signalling; and (5) no relevant effects on systemic inflammation. One may therefore speculate that HBO-induced stimulation of insulin sensitivity and mitochondrial function occurs at least partly via mitohormesis (i.e. low-dose ROS-mediated improvement of metabolic pathways) in type 2 diabetes.

## Supplementary information

ESM (PDF 531 kb)

## Data Availability

All data generated or analysed during this study are included in the published article (and its ESM). The files are available from the corresponding author upon reasonable request.

## Abbreviations

ATF: Activating transcription factor
BiP: Binding immunoglobulin protein
CON: Control condition (21% O_2_ ambient air delivered at 240 kPa compression)
cORP: Capacity oxidation reduction potential
CSA: Citrate synthase activity
DDZ: German Diabetes Center
EGP: Endogenous glucose production
eIF2α: Eukaryotic initiation factor 2α
FGF-21: Fibroblast growth factor-21
ER: Endoplasmic reticulum
GOX: Glucose oxidation
GSH: Free glutathione
GSSG: Oxidised glutathione
HBO: Hyperbaric oxygen (100% O_2_ delivered at 240 kPa compression)
iAUC: Incremental AUC
IHL: Intrahepatic lipid
IL-1ra: IL-1 receptor antagonist
IMCL: Intramyocellular lipid
LCR: Leak control ratio
LOX: Lipid oxidation
MPO: Myeloperoxidase
MRS: Magnetic resonance spectroscopy
NOXGD: Non-oxidative glucose disposal
P_i_: Inorganic phosphate
*R*_d_: Glucose disposal rate
REE: Resting energy expenditure
RER: Respiratory exchange ratio
ROS: Reactive oxygen species
SOD3: Superoxide dismutase 3
sORP: Static oxidation reduction potential
TBARS: Thiobarbituric acid reactive substances
tcpO_2_: Transcutaneous tissue oxygen
WAT: White adipose tissue
