# Supplementary material for: Hyperbaric oxygen rapidly improves tissue-specific insulin sensitivity and mitochondrial capacity in humans with type 2 diabetes: a randomised placebo-controlled crossover trial
Source: Diabetologia. 2022 Sep 30;66(1):57–69. doi: 10.1007/s00125-022-05797-0 (PMC9729133; doi:10.1007/s00125-022-05797-0)
Supplement: Supplementary file 1 — (PDF 531 kb) [file 125_2022_5797_MOESM1_ESM.pdf]

Extra Supplementary Material (Hyperbaric oxygen rapidly improves tissue-specific insulin sensitivity and mitochondrial capacity in humans with type 2 diabetes: a randomised placebo-controlled crossover trial)

ESM Table 1. Demographic and clinical characteristics of all study participants, given as means±SD or median (interquartile range).

Parameter	Means±SD or Median (IQR)
**n (% males)**	12 (100)
**Caucasian (%)**	100
**Age (years)**	58±7
**Body mass index (kg/m^2)**	30±3
**Waist circumference (cm)**	106±10
**Body fat (%)**	31±4
**Systolic blood pressure (mmHg)**	139±14
**Diastolic blood pressure (mmHg)**	89±9
**Known diabetes duration (years)**	5 (2)
**HbA_{1c} (mmol/mol) [%]**	53 (0.5) [7 (1)]
**M-value (mg*kg body weight^{-1}*min^{-1})**	3 (1)
**Fasting plasma triacylglycerol (mmol/l)**	9 (3)
**Fasting plasma non-esterified fatty acids (µmol/l)**	491 (205)
**Smoking status (%)**	0
**Use of metformin 1 g b. i. d. (%)**	100

ESM Table 2. Time course of finger pulse oximetry measuring blood oxygen saturation level (SpO_2) and transcutaneous tissue oxygen measurement measuring tissue oxygen level ($tcpO_2$), given as means±SD, during hyperbaric oxygen therapy (100% O_2, HBO) or ambient air therapy (21% O_2, control, CON) at -3 h (basal period), -1.5 h (intervention period) and 0 h (pre-clamp period) in humans with type 2 diabetes.

Parameter	Group	Time		
		**-3 h**	**-1.5 h**	**0 h**
**SpO_2 (%)**	CON	97±2	98±2	97±2
	HBO	97±2	100±0.4	99±1
**$tcpO_2$ (mmHg)**	CON	103±13	209±34	109±22
	HBO	101±18	1366±148***$^{\#\#}$	276±29*$^{\#}$

ANOVA adjusted for repeated measures within different time points of one intervention and between both interventions with Tukey-Kramer correction; n=16; *, 0.05 vs. CON of the same time point; ***, 0.001 vs. CON of the same time point; $^{\#}$, 0.05 vs. -3 h of the same group; $^{\#\#}$, 0.01 vs. -3 h of the same group.

ESM Table 3. List of parameters during the steady-state clamp period (+4.5 h - +5 h), given as means±SD, in humans with type 2 diabetes after hyperbaric oxygen (100% O_2, HBO) or ambient air treatment (21% O_2, control, CON) from -2.5 h – -0.5 h.

**Parameter**	**Group**	**Time**			
		**+4.5 h**	**+4.6 h**	**+4.8 h**	**+5 h**
**GIR ($mg*kg^{-1}*min^{-1}$)**	CON	2.6±1.2	2.6±1.2	2.6±1.2	2.5±1.2
	HBO	3.4±1.2	3.4±1.1	3.4±1.2	3.4±1.1
**[$^2H_2$]glucose APE (%)**	CON	2.7±0.1	2.7±0.1	2.7±0.1	2.7±0.1
	HBO	2.8±0.1	2.8±0.1	2.8±0.1	2.8±0.1

ANOVA adjusted for repeated measures within different time points of one intervention and between both interventions with Tukey-Kramer correction revealed no significant differences; n=10; GIR, glucose infusion rate; APE, atom percent enrichment.

ESM Table 4. Time course of respiratory exchange ratio (RER), resting energy expenditure (REE), glucose oxidation (GOX) and lipid oxidation (LOX) given as means±SD, during the basal (-24 h - -3 h), pre-clamp (0 h - +2 h) and clamp periods (+2 h – +5 h) in humans with type 2 diabetes after hyperbaric oxygen therapy (100% O_2, HBO) or ambient air therapy (21% O_2, control, CON) from -2.5 h - -0.5 h (intervention period).

Parameter	Group	Time		
		-23.5 h	+1.5 h	+4.5 h
**RER**	CON	0.85±0.05	0.85±0.09	0.86±0.06#
	HBO	0.84±0.07	0.83±0.11	0.87±0.05#
**REE (kJ/d)**	CON	7356±981	7396±1351	7503±1190#
	HBO	7311±893	7561±948	7699±1984#
**GOX ($mg*kg^{-1}*min^{-1}$)**	CON	1.25±0.08	1.30±0.22	2.05±0.73
	HBO	1.21±0.09	1.62±0.07*#	2.50±0.10*##
**LOX ($mg*kg^{-1}*min^{-1}$)**	CON	0.79±0.07	0.85±0.09	0.74±0.06
	HBO	0.81±0.18	0.60±0.08*#	0.56±0.04*#

ANOVA adjusted for repeated measures within different time points of one intervention and between both interventions with Tukey-Kramer correction; n=10; *, 0.05 vs. CON of the same time point; #, 0.05 vs. -23.5 h of the same group; ##, 0.01 vs. -23.5 h of the same group,

.

ESM Table 5. Time course of intrahepatocellular lipid (IHL) content, γ-adenosine triphosphate (ATP) and total inorganic phosphate (P_i) concentrations, as well as intramyocellular lipid content (IMCL) of m. tibialis anterior, given as means±SD, during the basal (-24 h - -3 h) and pre-clamp periods (0 h - +2 h) in humans with type 2 diabetes after hyperbaric oxygen therapy (100% O_2, HBO) or ambient air therapy (21% O_2, control, CON) from -2.5 h - -0.5 h (intervention period).

**Parameter**	**Group**	**Time**	
		**-24 h**	**+1 h**
**IHL ($CH_2/(CH_2+H_2O)$ in %)**	CON	8.6±0.2	8.3±0.1
	HBO	8.3±0.1	8.2±0.1
**ATP (mmol/l)**	CON	2.4±0.3	2.4±0.4
	HBO	2.2±0.5	3.6±0.6*$^{\#}$
**P_i (mmol/l)**	CON	1.8±0.5	1.9±0.4
	HBO	1.7±0.4	1.9±0.5
**IMCL (CH_2 (% of water resonance peak intensity)**	CON	0.27±0.001	0.28±0.001
	HBO	0.29±0.001	0.27±0.009

ANOVA adjusted for repeated measures within different time points of one intervention and between both interventions with Tukey-Kramer correction; n=9; *, 0.05 vs. CON of the same time point; $^{\#}$, 0.05 vs. -24 h of the same group.

ESM Table 6. Time course of serum thiobarbituric acid reactive substance (TBARS), oxidation reduction capacity and oxidation reduction potential, given as means±SD, during the basal (-24 h – -3 h), pre-clamp (0 h – +2 h) and clamp periods (+2 h – +5 h) in humans with type 2 diabetes after hyperbaric oxygen therapy (100% O_2, HBO) or ambient air therapy (21% O_2, control, CON) from -2.5 h – -0.5 h (intervention period).

**Variable**	**Group**	**Time**					
		**-24 h**	**0 h**	**+2 h**	**+3 h**	**+4 h**	**+5 h**
**TBARS (µmol/l)**	CON	0.66±0.56	0.60±0.37	0.50±0.37	0.48±0.28	0.43±0.35	0.41±0.35
	HBO	0.67±0.60	0.69±0.61	0.62±0.36	0.54±0.39	0.60±0.19	0.49±0.26
**Oxidation reduction capacity (µC)**	CON	0.10±0.02	0.11±0.01	0.11±0.02	0.11±0.02	n. d.	0.11±0.02
	HBO	0.11±0.02	0.11±0.01	0.11±0.01	0.10±0.02	n. d.	0.09±0.02
**Oxidation-reduction potential (mV)**	CON	174±8	174±6	166±10	167±8	n. d.	168±7
	HBO	176±11	174±8	173±7	174±8	n. d.	171±9

ANOVA adjusted for repeated measures within different time points of one intervention and between both interventions with Tukey-Kramer correction revealed no significant differences; n=12. n.d., not detectable.

ESM Table 7. Time course of myocellular endoplasmic reticulum stress markers, given as means±SD, during the basal (-24 h - -3 h) and pre-clamp periods (0 h - +2 h) in humans with type 2 diabetes after hyperbaric oxygen therapy (100% O_2, HBO) or ambient air therapy (21% O_2, control, CON) from -2.5 h - -0.5 h (intervention period).

Parameter	Group	Time	
		**-23 h**	**+0.5 h**
**ATF4 (AU)**	CON	1.83±0.81	1.75±0.58
	HBO	1.99±0.40	1.39±0.63
**ATF6 (AU)**	CON	1.33±0.83	1.59±1.69
	HBO	1.18±0.57	0.89±0.80
**BiP (AU)**	CON	1.11±0.50	1.53±0.41
	HBO	1.37±0.71	1.43±0.33
**eIF2α (AU)**	CON	1.03±0.40	1.17±0.28
	HBO	0.81±0.23	0.83±0.25
**p-eIF2α-S^{51} (AU)**	CON	0.52±0.25	0.65±0.03
	HBO	0.59±0.28	0.46±0.27

ANOVA adjusted for repeated measures within different time points of one intervention and between both interventions with Tukey-Kramer correction revealed no significant differences; n=10. ATF4, activating transcription factor 4; ATF6, activating transcription factor 6; AU, arbitrary units; BiP, binding immunoglobulin protein; eIF2α, eukaryotic initiation factor 2α; p-eIF2α-S^{51}, serine-51 phosphorylation of eIF2α.

ESM Table 8. Time course of myocellular insulin signaling, given as means±SD, during the basal (-24 h - -3 h), pre-clamp (0 h - +2 h) and clamp periods (+2 - +5 h) in humans with type 2 diabetes after hyperbaric oxygen therapy (100% O_2, HBO) or ambient air therapy (21% O_2, control, CON) from -2.5 h - -0.5 h (intervention period).

Parameter	Group	Time		
		-23 h	+0.5 h	+2.5
**IRS-1 (AU)**	CON	1.14±0.50	0.90±0.21	n.m.
	HBO	1.19±0.44	0.88±0.26	n.m.
**p-IRS-1-S^{1101} (AU)**	CON	0.91±0.28	1.69±0.42	n.m.
	HBO	0.89±0.29	1.19±0.23	n.m.
**p-IRS-1-S^{307} (AU)**	CON	0.85±0.24	0.96±0.28	n.m.
	HBO	0.98±0.35	0.73±0.33	n.m.
**Akt (AU)**	CON	1.12±0.44	1.16±0.73	1.51±0.73†
	HBO	0.86±0.35	0.80±0.34	1.40±0.84†
**p-Akt-S^{473} (AU)**	CON	0.95±0.38	1.02±0.44	1.52±0.42†
	HBO	0.99±0.30	1.14±0.28	2.01±0.36*†#
**p-Akt-T^{308} (AU)**	CON	0.90±0.31	0.87±0.34	1.12±0.32
	HBO	0.79±0.18	0.83±0.23	1.67±0.65*†#

ANOVA adjusted for repeated measures within different time points of one intervention and between both interventions with Tukey-Kramer correction; n=10; *, 0.05 vs. CON of the same time point; #, 0.05 vs. -24 h of the same group; †; 0.05 vs. +0.5h of the same group. AU, arbitrary units; IRS-1, insulin receptor substrate-1; p-IRS-1-S^{1101}, serine-1101 phosphorylation of IRS-1; p-IRS-1-S^{307}, serine-307 phosphorylation of IRS-1; Akt, protein kinase B; p-Akt-S^{473}, serine-473 phosphorylation of Akt; p-Akt-T^{308}, threonine-308 phosphorylation of AKT; n.m.; not measured.

ESM Table 9. Time course of plasma hormones and inflammatory markers, given as means±SD, during the basal (-24 h – +3 h), the pre-clamp (0 h - +2 h) and clamp periods (+2 h – +5 h) in humans with type 2 diabetes after hyperbaric oxygen therapy (100% O_2, HBO) or ambient air therapy (21% O_2, control, CON) from -2.5 h – -0.5 h (intervention period).

Variable	Group	Time					
		**-24 h**	**0 h**	**+2 h**	**+3 h**	**+4 h**	**+5 h**
**TNF-α (pg/ml)**	CON	1.3±0.21	1.7±0.62	1.3±0.30	1.0±0.11	1.0±0.12	0.9±0.10
	HBO	1.1±0.10	1.6±0.40	1.0±0.10	1.0±0.11	1.0±0.10	1.0±0.12
**IL-6 (pg/ml)**	CON	3.0±0.42	2.5±0.31	2.4±0.72	2.9±0.90	3.7±0.81	4.0±0.83
	HBO	3.8±0.44	3.2±0.80	3.5±0.90	4.9±0.83	5.0±1.0	5.3±0.70
**IL-1ra (pg/ml)**	CON	431±59	506±80	537±226	457±54	477±43	408±23
	HBO	569±91	521±76	500±64	478±58	552±69	529±77
**FGF-21 (pg/ml)**	CON	358±37	247±34	216±22	225±31	259±36	341±35
	HBO	393±59	227±32	187±21	191±24	259±27	370±47
**MPO (ng/ml)**	CON	231±49	259±52	227±45	167±30	217±42	167±29
	HBO	248±42	322±109	192±32	274±77	228±34	184±42
**SOD 3 (pg/ml)**	CON	9093±702	8568±769	8347±672	7242±409	6947±601	7178±998
	HBO	9318±910	8740±814	8742±813	7444±553	7784±766	8926±988
**Total adiponectin (ng/ml)**	CON	4882±638	4227±503	3740±387	4401±611	4267±557	4749±520
	HBO	4208±547	4114±381	4058±444	4108±485	3800±427	4133±413
**HMW adiponectin (ng/ml)**	CON	1859±440	1351±201	1256±171	1746±389	1503±236	2159±411
	HBO	1449±249	1512±267	1490±272	1657±380	1639±424	1519±275

ANOVA adjusted for repeated measures within different time points of one intervention and between both interventions with Tukey-Kramer correction revealed no significant differences; n=12; TNF-α, tumor necrosis factor alpha; IL-6, interleukin-6; IL-1ra, interleukin-1 receptor antagonist; FGF-21, fibroblast

growth factor-21; MPO, myeloperoxidase; SOD3, extracellular superoxide dismutase; HMW, high molecular weight.

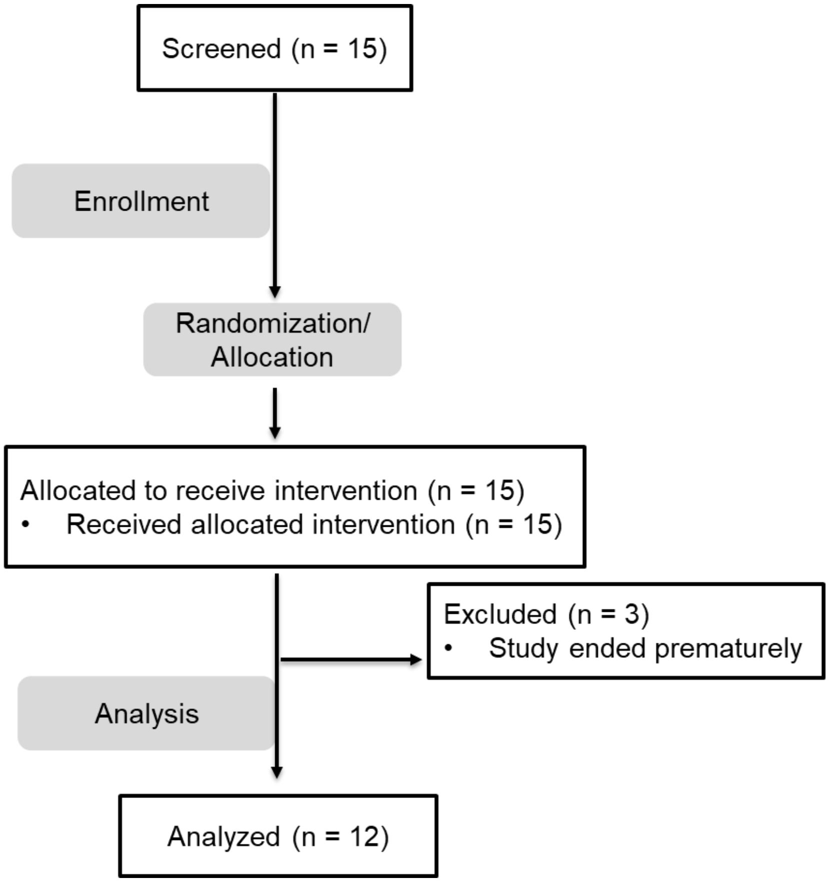

ESM Fig. 1: CONSORT flow diagram. Fifteen people with type 2 diabetes underwent screening, which included blood sampling, a medical history, clinical examination, anthropometry, electrocardiogram and bioimpedance analysis. All screened volunteers received the two interventions, but three volunteers were excluded prematurely. In total, 12 male participants completed the study and data were analyzed.

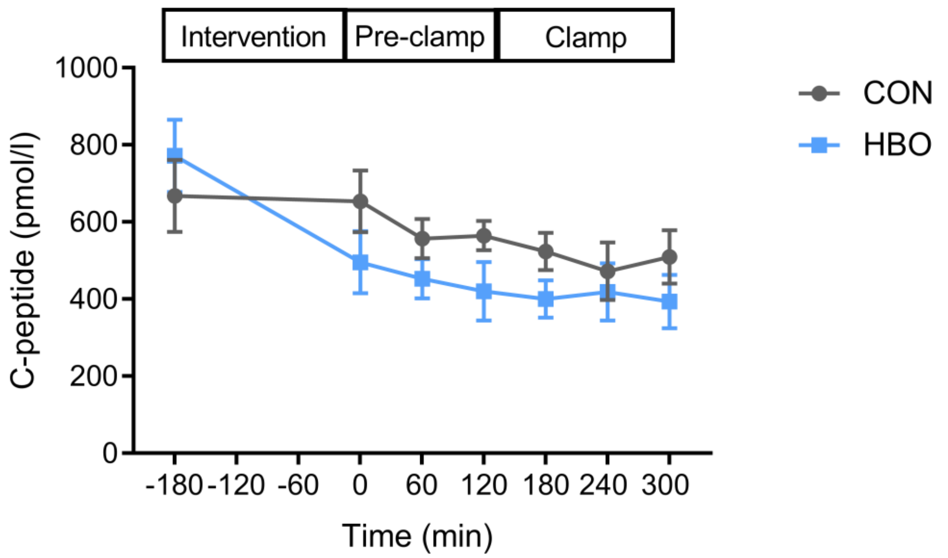

ESM Fig. 2: Time course of plasma C-peptide. Plasma C-peptide concentrations, given as means±SEM, in humans with type 2 diabetes (n=12) after two 2-hour sessions in a hyperbaric chamber with either 100% O_2 (HBO, blue color) or 21% O_2 ambient air (CON, grey color) from -2.5 h to -0.5 h (intervention period). Cross-over testing did not reveal any difference between the groups.
